# Longitudinal Anti-HBs Monitoring and Seroprotection Patterns in Rituximab-Treated Patients with Pemphigus Vulgaris: A Retrospective Cohort Study

**DOI:** 10.3390/diagnostics16183004

**Published:** 2026-09-17

**Authors:** Nur Ecer, Mehmet Harman

**Affiliations:** 1 Department of Dermatology and Venereology, Gazi Yaşargil Training and Research Hospital, 21070 Diyarbakır, Türkiye; 2 Department of Dermatology and Venereology, Faculty of Medicine, Dicle University, 21280 Diyarbakır, Türkiye

**Keywords:** anti-HBs, hepatitis B virus, pemphigus vulgaris, rituximab, seroprotection

## Abstract

Rituximab induces prolonged B-cell depletion and may alter protective antibody levels, but longitudinal antibody to hepatitis B surface antigen (anti-HBs) patterns in pemphigus vulgaris remain poorly characterized. This study evaluated anti-HBs levels and seroprotection after rituximab and explored associated factors. **Methods:** This retrospective cohort included 84 patients with pemphigus vulgaris treated with rituximab between 2010 and 2026 who had baseline and post-treatment anti-HBs measurements. Longitudinal changes in log-transformed anti-HBs levels were analyzed using a linear mixed-effects model with categorical time and a patient-specific random intercept. The probability of anti-HBs < 10 IU/L was assessed using a binomial generalized estimating equation model. Paired Wilcoxon signed-rank tests were performed as sensitivity analyses. **Results:** The median baseline anti-HBs level was 49.7 IU/L, and 34 patients (40.5%) had levels < 10 IU/L. Time was associated with longitudinal anti-HBs levels overall (*p* = 0.002). Compared with baseline, model-estimated levels were lower at month 6 (geometric mean ratio [GMR] 0.61, 95% CI 0.42–0.88), month 9 (GMR 0.64, 95% CI 0.45–0.89), and month 18 (GMR 0.50, 95% CI 0.32–0.78). Time was not significantly associated with the odds of anti-HBs < 10 IU/L (global *p* = 0.490). Positivity for antibody to hepatitis B core antigen (anti-HBc) IgG was associated with higher longitudinal anti-HBs levels (adjusted GMR 13.24, 95% CI 5.32–32.95; *p* < 0.001). No clinically or biochemically evident hepatitis B virus (HBV) reactivation was documented, although HBV DNA was not routinely monitored. **Conclusions:** Quantitative anti-HBs levels varied significantly during follow-up; whereas, the probability of crossing below the conventional seroprotection threshold did not increase significantly. These findings support HBV serological assessment and cautious interpretation of serial anti-HBs measurements in rituximab-treated patients with pemphigus vulgaris.

## 1. Introduction

Pemphigus vulgaris (PV) is a potentially life-threatening autoimmune blistering disease characterized by acantholysis involving the skin and mucous membranes. Its pathogenesis is primarily mediated by IgG autoantibodies directed against desmoglein 1 and desmoglein 3, transmembrane adhesion proteins expressed on the surface of keratinocytes [1,2].

Systemic corticosteroids remain an important component of PV treatment; however, their cumulative adverse effects and the occurrence of treatment-refractory disease have encouraged the use of steroid-sparing immunosuppressive therapies [3]. Rituximab, a chimeric monoclonal antibody targeting CD20-positive B lymphocytes, has become a first-line treatment for moderate-to-severe PV because of its ability to deplete autoreactive B-cell populations and provide sustained disease control [4,5].

The effects of rituximab on the human body are not limited to the suppression of pathogenic autoantibody production: rituximab-induced B-cell depletion disrupts humoral immunity more broadly, and may impair responses to vaccination as well as the maintenance of pre-existing protective antibodies [6]. This issue is particularly relevant in patients with previous hepatitis B virus (HBV) exposure or vaccine-induced immunity. HBV reactivation has also been reported in patients with PV receiving immunosuppressive treatment that included rituximab, emphasizing the importance of pretreatment HBV serological assessment [7].

Anti-hepatitis B surface antibodies (anti-HBs) are commonly used as a laboratory marker of pre-existing humoral immunity, and a concentration of ≥10 IU/L is conventionally considered indicative of seroprotection. Studies in patients with rheumatoid arthritis and hematological malignancies have shown that anti-HBs concentrations may change during rituximab-containing treatment and that baseline anti-HBs and anti-HBc antibody levels may provide clinically relevant information [8,9]. The risk associated with HBV exposure is particularly important when rituximab is administered together with systemic corticosteroids, as combined immunosuppression may further increase susceptibility to HBV reactivation [10].

Nevertheless, the interpretation of serial anti-HBs measurements during B-cell-depleting therapy remains challenging. Controlled vaccination studies have demonstrated impaired humoral responses in patients receiving rituximab [11], while longitudinal observations in autoimmune diseases indicate that B-cell depletion may have variable effects on established antibody-mediated immunity [12]. Prospective evidence from patients receiving rituximab-containing chemotherapy has also shown heterogeneous anti-HBs kinetics, with the greatest risk of antibody loss occurring among individuals with lower baseline concentrations [13].

A quantitative reduction in anti-HBs does not necessarily indicate that a patient has crossed below the conventional seroprotection threshold. Moreover, observed anti-HBs concentrations may be influenced by baseline immune status, previous natural infection or vaccination, concomitant immunosuppressive exposure, and the timing of laboratory measurements. Therefore, anti-HBs monitoring should be interpreted as a complementary assessment of pre-existing humoral immunity rather than as a substitute for established HBV screening and reactivation surveillance.

Patients with PV represent a clinically relevant population in which anti-HBs patterns may be shaped by B-cell depletion, systemic corticosteroid exposure, additional immunosuppressive treatment, and baseline HBV serological status. However, the longitudinal behavior and clinical interpretability of anti-HBs measurements after rituximab treatment have not been adequately characterized in this population. This study aimed to characterize the longitudinal behavior and clinical interpretability of anti-HBs as a laboratory marker of pre-existing humoral immunity in rituximab-treated patients with PV and to examine clinical and serological factors associated with quantitative anti-HBs levels and conventional seroprotection status.

## 2. Materials and Methods

### 2.1. Study Design and Setting

This retrospective cohort study was conducted at the Department of Dermatology and Venereology, Dicle University Faculty of Medicine Hospital. The electronic medical records of patients diagnosed with pemphigus vulgaris and evaluated between January 2010 and April 2026 were reviewed.

A total of 181 patient records were screened. The diagnosis of pemphigus vulgaris was based on compatible clinical findings together with histopathological examination, direct immunofluorescence, and/or serological testing, according to the information available in the medical records.

### 2.2. Study Population

Inclusion criteria: Patients were eligible when all of the following were met: (i) a diagnosis of PV established on compatible clinical findings together with histopathological examination, direct immunofluorescence, and/or serological testing, as documented in the medical records; (ii) treatment with at least one course of rituximab between January 2010 and April 2026; (iii) an available anti-HBs measurement obtained before the first rituximab infusion; and (iv) at least one available anti-HBs measurement after rituximab treatment.

**Figure 1:**
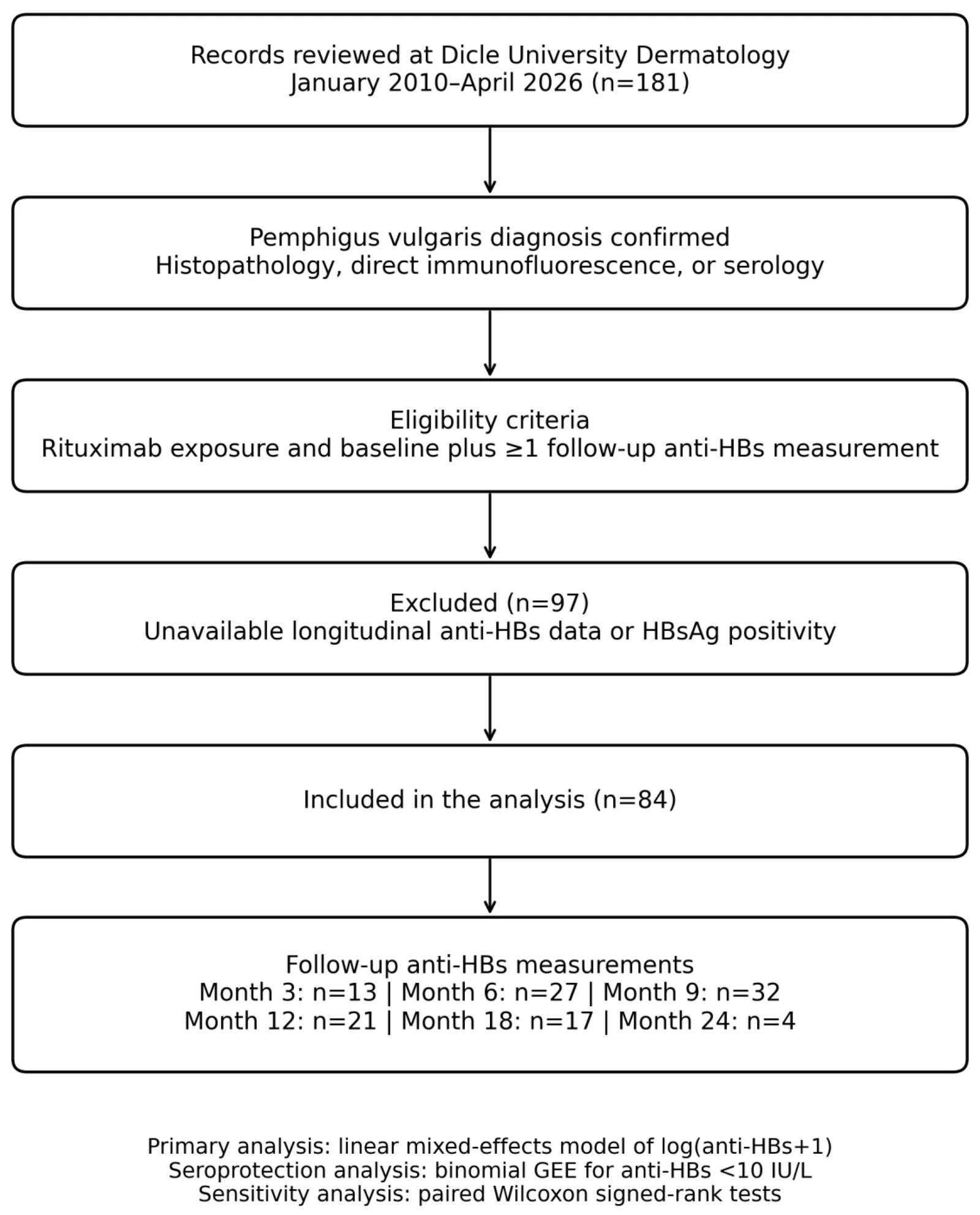
Patient selection, available follow-up measurements, and principal statistical analyses. HBsAg: hepatitis B surface antigen; anti-HBs: hepatitis B surface antibody; GEE: generalized estimating equation.

Exclusion criteria: Patients were excluded if they had (i) HBsAg positivity, indicating chronic hepatitis B virus infection; (ii) no baseline (pre-rituximab) anti-HBs measurement; or (iii) no post-rituximab anti-HBs measurement. Of the 181 screened records, 84 patients met all criteria and were included in the final analysis (Figure 1).

### 2.3. Data Collection

Demographic, clinical, treatment-related, and laboratory data were extracted from the electronic medical records. The variables collected included age, sex, disease duration, presence of systemic comorbidities, systemic corticosteroid use before and after rituximab, azathioprine use before and after rituximab, antiviral prophylaxis, isoniazid prophylaxis, baseline anti-HBc IgG status, anti-HBs levels, alanine aminotransferase levels, and aspartate aminotransferase levels. Rituximab was administered according to the rheumatoid arthritis protocol, consisting of two 1000 mg intravenous infusions given 15 days apart (days 0 and 15).

Anti-HBs and anti-HBc IgG were measured in the hospital’s central biochemistry laboratory by electrochemiluminescence immunoassay (ECLIA) on Roche cobas e analyzers, using the Elecsys Anti-HBs II (REF 08498610190) and Elecsys Anti-HBc II (REF 09014926190) reagent kits, respectively (Roche Diagnostics GmbH, Mannheim, Germany). According to laboratory records, the same Elecsys reagent kits were used throughout the study period; the analyzer platform was a cobas e 601 until 2025, after which the laboratory transitioned to a cobas e 801. Both are cobas e platforms running the same Elecsys ECLIA reagents, and results are reported on the same calibrator-traceable scale. Anti-HBs results were expressed in IU/L (numerically equivalent to the mIU/mL reported by the assay), and a concentration ≥10 IU/L was taken to indicate seroprotection.

In accordance with institutional practice, baseline anti-HBs and anti-HBc IgG were obtained as part of the mandatory pre-rituximab screening work-up and therefore preceded the first rituximab infusion in all patients. Because sampling dates were not systematically retrievable from the archived records, the interval between baseline serological sampling and the first infusion could not be quantified.

Anti-HBs measurements were evaluated at baseline and at the available follow-up windows corresponding to months 3, 6, 9, 12, 18, and 24 after rituximab treatment. Because only four patients had a month-24 measurement, the pre-specified analysis window for all inferential analyses was baseline through month 18; month 24 is reported descriptively only. Because the study was retrospective, anti-HBs was not measured at protocolized fixed intervals, and laboratory measurements were not available for every patient at every nominal follow-up window. All 84 included patients contributed a baseline and at least one post-rituximab measurement; the number of patients contributing to each individual time point therefore varies and does not represent loss to follow-up. A total of 194 anti-HBs measurements from 84 patients were available through month 18 and were included in the longitudinal models.

### 2.4. Outcomes

The primary outcome was longitudinal change in quantitative anti-HBs levels following rituximab treatment. Anti-HBs concentrations were analyzed as continuous measurements.

The principal secondary outcome was the longitudinal probability of having an anti-HBs level below 10 IU/L. An anti-HBs concentration of ≥10 IU/L was considered to represent the conventional seroprotective threshold; whereas, values < 10 IU/L were classified as being below this threshold.

Additional exploratory outcomes included the associations of baseline anti-HBc IgG status, age, sex, disease duration, comorbidity, and previous azathioprine exposure with longitudinal anti-HBs levels. Liver enzyme results and clinically documented HBV reactivation events were also reviewed.

HBV reactivation was not treated as a formal study outcome. HBV DNA testing by polymerase chain reaction was not performed systematically during follow-up and was not part of the study protocol; accordingly, no PCR platform, assay, or limit of detection is reported. Reactivation was evaluated only as clinically or biochemically evident events documented in the medical records (that is, a recorded clinical diagnosis of reactivation and serial serum aminotransferase levels). Consequently, the absence of clinically or biochemically evident reactivation could not exclude subclinical or exclusively virological HBV reactivation.

### 2.5. Statistical Analysis

Categorical variables are presented as n (%) and continuous variables as mean ± SD or median (IQR). Because anti-HBs concentrations were markedly right-skewed, they were analyzed as log(anti-HBs + 1). Values reported by the laboratory as below the assay’s lower limit of quantification (<2 mIU/mL) were assigned a value of 1 IU/L before transformation; because the primary analysis modeled log(anti-HBs + 1), this substitution affects only the extreme left tail of the distribution and does not alter the ranking of observations used in the non-parametric sensitivity analyses.

The primary longitudinal analysis used a linear mixed-effects model with log(anti-HBs + 1) as the dependent variable, follow-up time as a categorical fixed effect (baseline as reference), and a patient-specific random intercept to account for within-patient correlation and the unbalanced number of observations. Coefficients were estimated by restricted maximum likelihood, exponentiated, and reported as geometric mean ratios (GMR) with 95% CIs; the global effect of time was tested by a likelihood-ratio test comparing maximum-likelihood-refitted nested models, and baseline-versus-follow-up comparisons were adjusted using the Holm procedure. An exploratory adjusted model additionally included baseline anti-HBc IgG status, age, sex, disease duration, comorbidity, and prior azathioprine use, with age and disease duration standardized (estimates per 1-standard-deviation increase).

The longitudinal probability of anti-HBs < 10 IU/L was modeled using a binomial generalized estimating equation (logit link, exchangeable working correlation) with categorical time and baseline anti-HBc IgG status, reported as odds ratios with 95% CIs; the global effect of time was assessed by a Wald chi-square test. Antiviral prophylaxis was not entered as an independent predictor because of near-complete overlap with anti-HBc IgG positivity (32 of 33 prophylaxis recipients were anti-HBc IgG-positive, and 32 of 33 anti-HBc IgG-positive patients received prophylaxis), which would have introduced substantial collinearity and confounding by indication. As sensitivity analyses, Holm-adjusted paired Wilcoxon signed-rank tests (effect size r = |Z|/√n) compared baseline with months 3, 6, 9, 12, and 18 among patients with both measurements available.

Missing values were not imputed; all available measurements through month 18 (194 measurements from 84 patients) contributed to the longitudinal models under a missing-at-random assumption. Month 24 was excluded from all inferential analyses because only four measurements were available—it is reported descriptively. Model assumptions were assessed by inspection of residual and quantile–quantile plots.

All tests were two-sided and *p* < 0.05 was considered significant. Analyses were performed in Python version 3.13.5 using statsmodels version 0.14.6 and SciPy version 1.17.0.

## 3. Results

### 3.1. Demographic and Clinical Characteristics

The study included 84 patients with pemphigus vulgaris who met the eligibility criteria. Patient selection is shown in Figure 1, and baseline demographic and clinical characteristics are summarized in Table 1.

The mean age was 50.1 ± 15.0 years (range, 17–87 years), and 62 patients (73.8%) were female. Mean disease duration was 7.6 ± 4.7 years. At least one systemic comorbidity was present in 19 patients (22.6%), most commonly diabetes mellitus, hypertension, or thyroid disease.

All patients had received systemic corticosteroids before rituximab, and 82 (97.6%) continued corticosteroid treatment after rituximab. Azathioprine had been used by 62 patients (73.8%) before rituximab and by 46 (54.8%) afterward. Antiviral prophylaxis was administered to 33 patients (39.3%), and baseline anti-HBc IgG positivity was present in 33 (39.3%). Baseline HBV serology is summarized in Table 2. Anti-HBc IgG positivity was strongly associated with baseline seroprotection: 30 of 33 anti-HBc IgG-positive patients (90.9%) had anti-HBs ≥ 10 IU/L, compared with 20 of 51 anti-HBc IgG-negative patients (39.2%).

### 3.2. Descriptive and Paired Changes in Anti-Hbs Levels

Baseline anti-HBs measurements were available for all 84 patients. Follow-up measurements were available for 13 patients at month 3, 27 at month 6, 32 at month 9, 21 at month 12, 17 at month 18, and four at month 24, as summarized in Figure 1.

The overall median baseline anti-HBs level was 49.7 IU/L (IQR, 1.0–481.3), and 34 patients (40.5%) had values below 10 IU/L. Crude follow-up medians ranged from 13.5 to 65.7 IU/L. Because different subsets of patients contributed data at each visit, these crude values were treated as descriptive and were not interpreted as a continuous trajectory of an identical cohort.

In paired sensitivity analyses, the median within-patient change was −32.1 IU/L at month 3, −14.1 IU/L at month 6, −0.5 IU/L at month 9, −6.1 IU/L at month 12, and −19.5 IU/L at month 18. Effect sizes were moderate to large (r = 0.48–0.79), and all five comparisons remained statistically significant after Holm adjustment. The descriptive and paired findings are presented in Table 3.

The proportion of patients with anti-HBs < 10 IU/L was 30.8% at month 3, 40.7% at month 6, 50.0% at month 9, 33.3% at month 12, 47.1% at month 18, and 50.0% at month 24 (Table 3). Month 24 findings were reported descriptively only because only four patients had available measurements.

### 3.3. Longitudinal Mixed-Effects and Seroprotection Models

**Figure 2:**
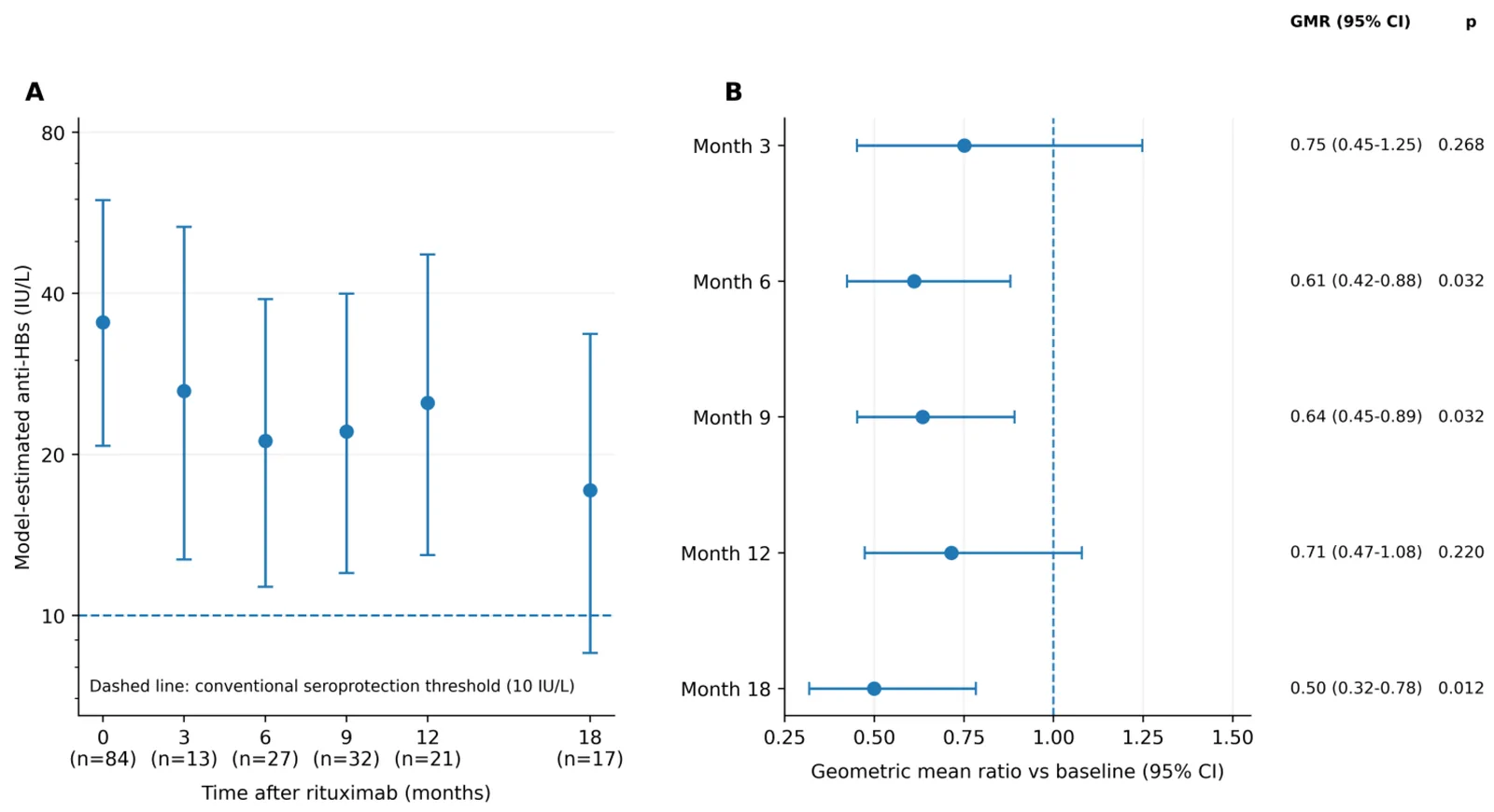
Model-based longitudinal anti-HBs results. (**A**) Back-transformed fixed-effect estimates from the linear mixed-effects model of log(anti-HBs + 1); error bars indicate 95% confidence intervals, and the *x*-axis labels state the number of available observations contributing at each time point. The dashed horizontal line indicates the conventional seroprotection threshold of 10 IU/L. (**B**) Geometric mean ratios relative to baseline with 95% confidence intervals and Holm-adjusted *p* values. Month 24 was excluded from inferential modeling because only four measurements were available.

The mixed-effects model included 194 anti-HBs measurements through month 18. Time was significantly associated with log-transformed anti-HBs levels overall (likelihood-ratio χ^2^ = 18.73, 5 df; *p* = 0.002). After Holm adjustment, model-estimated anti-HBs + 1 levels were lower than baseline at month 6 (GMR 0.61, 95% CI 0.42–0.88; adjusted *p* = 0.032), month 9 (GMR 0.64, 95% CI 0.45–0.89; adjusted *p* = 0.032), and month 18 (GMR 0.50, 95% CI 0.32–0.78; adjusted *p* = 0.012). Differences at months 3 and 12 were not statistically significant. Full model estimates are reported in Table 4; model-estimated anti-HBs levels are shown in Figure 2A, and geometric mean ratios relative to baseline are shown in Figure 2B.

In the anti-HBc-adjusted GEE model, the global effect of time on the odds of anti-HBs < 10 IU/L was not statistically significant (Wald χ^2^ = 4.43, 5 df; *p* = 0.490). No individual follow-up time point showed a statistically significant increase in the odds of being below the conventional seroprotective threshold (Table 4).

### 3.4. Clinical and Serological Factors Associated with Anti-Hbs Levels

In the exploratory adjusted mixed model, baseline anti-HBc IgG positivity was associated with substantially higher anti-HBs + 1 levels (adjusted GMR 13.24, 95% CI 5.32–32.95; *p* < 0.001). Age, sex, disease duration, comorbidity, and previous azathioprine use were not independently associated with longitudinal anti-HBs levels (Table 4).

Antiviral prophylaxis and anti-HBc IgG status showed near-complete overlap: 32 of 33 patients receiving antiviral prophylaxis were anti-HBc IgG-positive, and 32 of 33 anti-HBc IgG-positive patients received prophylaxis. Antiviral prophylaxis was therefore not evaluated as an independent determinant, and unadjusted differences according to prophylaxis were interpreted as confounding by indication.

In the GEE model, anti-HBc IgG positivity was associated with lower odds of anti-HBs < 10 IU/L (OR 0.10, 95% CI 0.03–0.29; *p* < 0.001; Table 4). This finding reflects higher pre-existing antibody concentrations and should not be interpreted as protection from HBV reactivation.

### 3.5. Liver Enzymes and Hbv Reactivation

Median ALT levels remained between 12.0 and 18.0 U/L from baseline through month 18; whereas, median AST levels remained between 17.5 and 20.0 U/L, as summarized in Table 5.

No marked transaminase elevation suggesting clinically evident HBV reactivation was documented, and no clinically recorded or biochemically evident HBV reactivation occurred. However, because HBV DNA was not routinely measured, subclinical or exclusively virological reactivation could not be excluded.

## 4. Discussion

This retrospective cohort study characterized longitudinal anti-HBs patterns in patients with pemphigus vulgaris treated with rituximab. The mixed-effects analysis demonstrated a significant overall effect of time, with lower model-estimated anti-HBs levels at months 6, 9, and 18. In contrast, the probability of anti-HBs levels falling below the conventional seroprotective threshold of 10 IU/L did not increase significantly over time. This distinction is clinically relevant, as a quantitative reduction in antibody levels does not necessarily translate into categorical loss of seroprotection.

Previous studies in rheumatologic and hematologic populations have reported reductions in anti-HBs levels after rituximab exposure. Ulutaş et al. observed anti-HBs decline in patients with rheumatoid arthritis receiving rituximab, while Araujo-Neto et al. reported loss of detectable antibodies in a subset of patients receiving rituximab-containing chemotherapy, particularly among those with lower baseline antibody levels [8,13]. Our findings extend these observations to patients with pemphigus vulgaris, a population in which longitudinal anti-HBs data remain limited.

Anti-HBc IgG positivity was associated with substantially higher longitudinal anti-HBs levels and lower odds of anti-HBs levels below 10 IU/L. This association most likely reflects previous natural HBV exposure and higher pre-existing antibody concentrations. The relative persistence of established antibody titers during CD20-directed therapy is biologically consistent with the compartmentalization of humoral memory. Rituximab depletes CD20-expressing B cells, but terminally differentiated antibody-secreting plasma cells no longer express CD20 and are therefore not directly targeted; in patients treated with rituximab, such cells have been shown to persist as long-lived plasma cells within tissue survival niches and to continue secreting antibody independently of the circulating B-cell pool [14,15,16]. Pre-existing, vaccine- or infection-induced anti-HBs may thus be maintained for prolonged periods despite profound peripheral B-cell depletion; whereas, the generation of new antibody responses, which requires activation and differentiation of naive and memory B cells, is considerably more vulnerable, as demonstrated in controlled vaccination studies in rituximab-treated patients [11]. This dissociation between preserved established immunity and impaired de novo responses may partly explain why quantitative anti-HBs declined in our cohort without a corresponding increase in the odds of crossing below the seroprotective threshold. However, this finding should not be interpreted as evidence that anti-HBc positivity protects against HBV reactivation. Patients with resolved HBV infection remain at increased risk of reactivation during B-cell-depleting therapy [14,15].

Antiviral prophylaxis showed near-complete overlap with anti-HBc IgG positivity. Accordingly, the higher anti-HBs levels observed among patients receiving prophylaxis likely reflect confounding by indication rather than an independent effect of antiviral treatment on antibody concentrations. Antiviral prophylaxis was therefore not evaluated as an independent determinant in the adjusted models. This interpretation is consistent with current recommendations supporting nucleos(t)ide analog prophylaxis in patients at high risk of HBV reactivation during rituximab therapy [17,18].

No clinically or biochemically evident HBV reactivation was documented. Nevertheless, HBV DNA was not routinely monitored, and stable aminotransferase levels cannot exclude subclinical or exclusively virological reactivation. The present study should therefore be interpreted as an analysis of anti-HBs and seroprotection patterns rather than as an assessment of HBV reactivation incidence. Rituximab carries a recognized risk of HBV reactivation, underscoring the importance of pretreatment screening and appropriate monitoring [19].

The observed antibody changes cannot be attributed exclusively to rituximab. All patients had received systemic corticosteroids, nearly all continued corticosteroid therapy after rituximab, and a substantial proportion had also received azathioprine. The findings therefore reflect the combined effects of B-cell depletion, cumulative immunosuppressive exposure, baseline immune status, and other unmeasured clinical factors.

The strengths of this study include the evaluation of a relatively large real-world pemphigus vulgaris cohort, the availability of baseline anti-HBs measurements for all participants, and the use of mixed-effects and generalized estimating equation models to account for unbalanced repeated measurements. The simultaneous assessment of anti-HBc IgG status, antiviral prophylaxis, and liver enzyme levels also provided clinically relevant context.

Several limitations should be acknowledged. The retrospective, single-center design limited control of residual confounding. Because anti-HBs was not re-tested at protocolized fixed intervals in routine practice, the number of patients contributing measurements differed substantially between time points, with only four patients evaluated at month 24; although all included patients contributed at least one post-rituximab measurement and the longitudinal models used all available observations under a missing-at-random assumption, estimates at the more sparsely sampled later time points should be interpreted with caution. The interval between baseline anti-HBs sampling and the first rituximab infusion could not be quantified retrospectively; although baseline serology preceded rituximab in all patients as part of pre-treatment screening, variation in this interval cannot be excluded and represents a constraint on reproducibility. Laboratory measurements were performed over a 16-year period, during which the analyzer platform changed (cobas e 601 to cobas e 801 in 2025) while the Elecsys ECLIA reagent kits and reporting units remained unchanged; although both platforms are calibrator-traceable and report results on the same scale, minor inter-platform variability cannot be entirely excluded, but because anti-HBs was analyzed longitudinally within patients and the transition occurred late in the study period, such an effect is unlikely to account for the time-related changes observed at months 6, 9, and 18. Vaccination histories were unavailable, preventing distinction between vaccine-induced and infection-induced immunity. Concomitant immunosuppressive burden could not be fully standardized. In addition, the near-complete overlap between antiviral prophylaxis and anti-HBc IgG positivity precluded independent evaluation of these variables. Finally, the absence of routine HBV DNA monitoring prevented exclusion of subclinical or exclusively virological reactivation.

These findings support comprehensive HBV serological assessment before rituximab initiation. Anti-HBs measurement may provide complementary information regarding pre-existing humoral immunity but should not replace guideline-recommended HBsAg and anti-HBc screening, HBV DNA monitoring when indicated, or antiviral prophylaxis in patients at risk [17,18,19]. Prospective studies incorporating standardized follow-up intervals, vaccination history, serial HBV DNA testing, and detailed treatment exposure are needed to clarify the clinical value of serial anti-HBs monitoring in patients with pemphigus vulgaris.

## 5. Conclusions

Rituximab-treated patients with PV showed significant longitudinal variation in anti-HBs levels, with lower model-estimated levels at months 6, 9, and 18. However, the likelihood of falling below the conventional seroprotective threshold did not increase significantly over time. Anti-HBc IgG positivity was associated with higher anti-HBs levels. In practical terms, serial anti-HBs measurement may serve as an adjunctive laboratory marker of pre-existing humoral immunity and may help identify patients with low baseline titers who warrant closer attention; it should not, however, be regarded as a surrogate for—or a substitute for—guideline-recommended pretreatment HBsAg and anti-HBc screening, HBV DNA monitoring where indicated, and antiviral prophylaxis in at-risk patients. Prospective studies with standardized monitoring intervals and serial HBV DNA measurements are needed to define the clinical value of serial anti-HBs monitoring in this population.

## Figures and Tables

**Table 1:** Baseline demographic and clinical characteristics of the study population (*n* = 84).

Parameter	Value (n = 84)
Age (years), mean ± SD	50.1 ± 15.0
Age, median (min–max)	48 (17–87)
Sex, female/male, n (%)	62 (%73.8)/22 (%26.2)
Disease duration (years), mean ± SD	7.6 ± 4.7
Disease duration, median (min–max)	6.5 (1–20)
Presence of comorbidity, n (%)	19 (%22.6)
Systemic steroid use before RTX, n (%)	84 (%100.0)
Systemic steroid use after RTX, n (%)	82 (%97.6)
Azathioprine use before RTX, n (%)	62 (%73.8)
Azathioprine use after RTX, n (%)	46 (%54.8)
Antiviral prophylaxis, n (%)	33 (%39.3)
Isoniazid (INH) prophylaxis, n (%)	18 (%21.4)
Baseline anti-HBc IgG positivity, n (%)	33 (%39.3)

**Table 2:** Baseline HBV serological profile of the study population (n = 84).

Parameter	Value (n = 84)
HBsAg positive, n (%)	0 (0.0) *
Anti-HBc IgG positive, n (%)	33 (39.3)
Anti-HBc IgG negative, n (%)	51 (60.7)
Anti-HBs ≥ 10 IU/L, n (%)	50 (59.5)
Anti-HBs < 10 IU/L, n (%)	34 (40.5)
Anti-HBs (IU/L), median (IQR)	49.7 (1.0–481.3)
Anti-HBc IgG positive: anti-HBs ≥ 10 IU/L, n (%)	30 (90.9)
Anti-HBc IgG positive: anti-HBs < 10 IU/L, n (%)	3 (9.1)
Anti-HBc IgG negative: anti-HBs ≥ 10 IU/L, n (%)	20 (39.2)
Anti-HBc IgG negative: anti-HBs < 10 IU/L, n (%)	31 (60.8)
Antiviral prophylaxis, n (%)	33 (39.3)
Isoniazid (INH) prophylaxis, n (%)	18 (21.4)

* HBsAg positivity was an exclusion criterion; no included patient had chronic HBV infection. Seroprotection threshold, ≥10 IU/L. Percentages in the cross-classified rows are calculated within the corresponding anti-HBc IgG stratum. Anti-HBs and anti-HBc IgG were measured by electrochemiluminescence immunoassay (Elecsys Anti-HBs II and Elecsys Anti-HBc II; Roche cobas e analyzers).

**Table 3:** Descriptive and paired changes in anti-HBs levels during follow-up.

Time Point	n	Observed Median (IQR)	<10 IU/L, n (%)	Paired Baseline Median (Iqr)	Median Change	Effect Size r	Holm-Adjusted *p*
Month 0	84	49.7 (1.0–481.3)	34 (40.5%)	—	—	—	—
Month 3	13	65.7 (1.0–680.7)	4 (30.8%)	152.3 (2.1–832.1)	−32.1	0.70	0.019
Month 6	27	39.1 (1.0–111.9)	11 (40.7%)	71.8 (1.0–569.2)	−14.1	0.72	<0.001
Month 9	32	13.5 (1.0–76.5)	16 (50.0%)	41.8 (1.0–645.7)	−0.5	0.48	0.019
Month 12	21	36.8 (2.0–330.3)	7 (33.3%)	66.9 (1.0–608.3)	−6.1	0.56	0.019
Month 18	17	15.1 (1.0–98.6)	8 (47.1%)	70.5 (2.5–272.7)	−19.5	0.79	0.005
Month 24	4	37.4 (1.0–190.3)	2 (50.0%)	333.0 (10.7–690.3)	−138.9	—	Descriptive only

Values at follow-up are based on patients with both baseline and corresponding follow-up measurements. Median change was calculated as follow-up minus baseline. Effect size r = |Z|/√n. Month 24 was not tested because only four measurements were available.

**Table 4:** Linear mixed-effects and GEE model results.

Predictor	LMM GMR (95% CI)	*p *Value *	GEE OR for Anti-HBs < 10 (95% CI)	*p *Value
Month 3 vs. baseline	0.75 (0.45–1.25)	0.268	1.07 (0.90–1.27)	0.465
Month 6 vs. baseline	0.61 (0.42–0.88)	0.032	1.46 (0.95–2.23)	0.081
Month 9 vs. baseline	0.64 (0.45–0.89)	0.032	1.18 (0.75–1.85)	0.477
Month 12 vs. baseline	0.71 (0.47–1.08)	0.220	1.01 (0.88–1.16)	0.865
Month 18 vs. baseline	0.50 (0.32–0.78)	0.012	1.57 (0.89–2.78)	0.122
anti-HBc positive	13.24 (5.32–32.95)	<0.001	0.10 (0.03–0.29)	<0.001
Age (per SD)	0.89 (0.56–1.41)	0.613	—	—
Female	1.50 (0.55–4.09)	0.432	—	—
Disease duration (per SD)	0.85 (0.54–1.35)	0.486	—	—
Comorbidity	0.43 (0.15–1.29)	0.132	—	—
Prior azathioprine	0.50 (0.18–1.41)	0.191	—	—

* Time-point *p* values are Holm-adjusted; covariate p values are unadjusted. GMRs refer to anti-HBs + 1. The GEE included time and baseline anti-HBc status. Antiviral prophylaxis was excluded because 32 of 33 prophylaxis recipients were anti-HBc-positive.

**Table 5:** Median ALT and AST levels during the follow-up period.

Parameter	Month 0	Month 3	Month 6	Month 9	Month 12	Month 18
ALT (U/L), median	18.0	17.0	16.0	15.0	15.0	12.0
AST (U/L), median	18.0	18.0	20.0	17.5	20.0	20.0
n (measurements)	84	13	26–27	32	21	17

Because the number of measurements at the 24th month (n = 4) was limited, this time point was not included in the table. The number of measurements for ALT and AST at the 6th month (27 and 26, respectively) differs by one patient.

## Data Availability

The datasets generated and/or analyzed during the current study are available from the corresponding author on reasonable request, subject to institutional and ethical restrictions.
